# Daoyin therapy in chronic neck pain: study protocol for a randomized controlled trial

**DOI:** 10.1186/s12906-024-04386-5

**Published:** 2024-03-15

**Authors:** Xiangxu Chen, Mingze Zhu, Wei Li, Daan Wang, Jing Liu

**Affiliations:** 1 Department of Traditional Chinese Sports and Health, College of Martial Arts, Shanghai University of Sport, No. 399 Changhai Street, Shanghai City, China; 2 Department of Occupational and Environmental Health, Hudson College of Public Health, University of Oklahoma Health Sciences Center, Oklahoma City, OK 73104 USA; 3 Department of Athletic Training and Instruction, College of Graduate, Guangzhou Sport University, No. 1268 Guangzhou Avenue Central, Guangzhou City, China; 4 Department of Physical Education and Health, Hainan Tropical Ocean University, No.1 Yucai Street, Sanya City, China

**Keywords:** Daoyin therapy, Chronic neck pain, Protocol, Randomized controlled trial

## Abstract

**Background:**

Daoyin therapy (DT), an ancient therapeutic approach with a history spanning thousands of years, has traditionally been employed to address musculoskeletal pain and psychosomatic disorders. However, the application of DT for chronic neck pain (CNP) has received limited attention in the existing literature, and systematic randomized clinical trials (RCTs) in this context remain scarce. This manuscript outlines an RCT protocol designed to investigate whether DT is more effective at alleviating CNP in adult individuals compared to other interventions.

**Methods:**

A 12-week RCT was conducted, with participants undergoing randomization into one of three groups: DT, Meditation + Fitness Exercise (M+FE), or a control group. Participants in the DT and M + FE groups attended their respective training classes three times per week for 12 weeks. Participants in the control group were required to attend health education workshops every 2 weeks. Following the 12-week intervention period, all participants underwent follow-up assessments at the 16th week. Outcome measures encompassed the Simplified Chinese Neck Pain and Disability Scale (SC-NPAD) and Visual Analog Scale (VAS) for pain assessment, Static Neck Posture Assessment (SNPA) to evaluate neck and shoulder posture and function, Short Form-36 (SF-36) to assess quality of life, and blood tests measuring 5-Hydroxytryptamine (5-HT), Norepinephrine/Noradrenaline (NE/NA), γ-aminobutyric acid (GABA), Adreno-Cortico-Tropic-Hormone (ACTH), β-Endorphin (β-EP), and Calcitonin-Gene-Related Peptide (CGRP) levels via high-performance liquid chromatography (HPLC), chemiluminescence immunoassay (CLIA), enzyme-linked immunosorbent assay (ELISA), and radioimmunoassay (RIA). Brain activity changes were monitored through MRI scans. Repeated measures analyses of variance (ANOVAs) will be used to evaluate the outcomes at baseline, at the 12th week, and at the 16th week. Generalized Estimating Equation (GEE) models will be applied to analyze changes in outcomes over time and differences between groups.

**Discussion:**

This trial aims to evaluate the efficacy of DT in comparison to other interventions and explore the neuroendocrine mechanisms underlying its effects in adults with CNP. If the intervention and procedures demonstrate feasibility and acceptability, there are plans to conduct a more extensive controlled trial. This could potentially pave the way for the broader application of DT, not only in the context of CNP but also for other chronic diseases.

**Trial registration:**

This trial has been registered with the Chinese Clinical Trial Registry (Registration ID: [ChiCTR2400079571]).

## Background

Chronic neck pain (CNP) constitutes a significant global public health and socioeconomic concern, impacting a substantial portion of the population across their lifespan [1]. The lifetime prevalence of CNP is estimated to be around 50%, and it imposes significant societal and individual burdens [2]. Recent investigations have demonstrated a wide-ranging overall prevalence of CNP, spanning from 0.4% to 86.8% (with an average of 23.1%). Notably, within populations at high risk for CNP, such as sedentary workers, the one-year CNP incidence ranges from 10.4% to 21.3% [3], surpassing the rates observed among blue-collar workers.

This high prevalence and incidence of CNP has spurred research into the underlying mechanisms and neural correlates of chronic pain. Over the years, in the field of neuroimaging studies, the prevailing focus has leaned towards functional connectivity (FC) trials, predominantly utilizing the insula as a pivotal seed point. These endeavors collectively shed light on the disrupted FC between the insula and the default network in individuals afflicted by chronic pain. This disruption underscores the intricate interplay between pain processing, perceptual recognition, and cognitive and emotion-related brain region engagement, thus serving as a foundational basis for delving into central neural aspects of chronic pain [4, 5]. However, the intricate nexus between distinct chronic pain subtypes and their corresponding brain regions introduces a nuanced complexity. A plethora of studies have revealed abnormal activation patterns across diverse brain regions and disruptions in FC among chronic pain patients [4, 6]. 

In the present landscape, functional magnetic resonance imaging (fMRI) methodologies predominantly illuminate brain gray matter dynamics in conditions such as lower back pain, back pain, tension-type headaches, and fibromuscular pain syndrome. These examinations have unequivocally exposed structural modifications within brain gray matter as an accompaniment to chronic pain. However, pertinent research focusing on chronic skeletal muscle pain has presented a distinctive narrative. In a study encompassing 13 patients, no discernible differences in gray matter distribution within brain regions were observed when contrasted with normal subjects [7]. Nevertheless, an augmentation in gray matter volume surfaced in prefrontal and somatosensory regions subsequent to an 11-week cognitive-behavioral intervention [7]. Consequently, the intricate relationship between neural activity within specific brain regions and gray matter configurations in the context of chronic pain, including CNP, necessitates further elucidation.

Additionally, there is compelling evidence linking the neuroendocrine system with pain perception. The N-methyl-D-aspartic acid receptor (NMDA) in the rostral ventromedial medulla (RVM) plays a key role in pain modulation, with receptor phosphorylation leading to enhanced channel conductance and increased synaptic insertion of NMDA receptors [8, 9]. Studies reveal that serotonin injection or inhibiting the serotonin transporter (SERT) in the RVM offers analgesic effects, and blocking serotonin receptors hinders analgesia from periaqueductal gray (PAG) stimulation, while systemic morphine boosts serotonin levels in the RVM [10, 11]. During physical activity, opioid receptor activation curbs NMDA receptor phosphorylation and lowers serotonin transporter expression, ultimately decreasing pain sensitivity [12]. Regular physical activity, by activating opioid and serotonin pathways, thus acts as a preventive measure against heightened pain sensitivity.

As we explore interventions for CNP, exercise therapy emerges as a promising intervention for alleviating CNP, displaying consistent benefits across various exercise modalities, be it isometric or isotonic neck strengthening or endurance exercises [13]. Conversely, the impact of stretching exercises appears more constrained in its efficacy [14]. Furthermore, complementary approaches from the realm of alternative medicine, encompassing practices such as Yoga and Qigong, have garnered attention for their notable effectiveness in addressing neck pain [15–17]. These alternative exercise modalities furnish patients with viable options that deviate from conventional exercises.

Daoyin therapy (DT), rooted in the principles of Traditional Chinese Medicine (TCM), encompasses dynamic musculoskeletal training, synchronized breathing techniques, and meditative practices. TCM functional exercises like Taijiquan, Baduanjin, and Liuzijue all fall under the purview of DT [18], embodying its core tenets. Recognized for its health-enhancing attributes, DT has garnered mounting empirical [19] support for its potential to ameliorate conditions such as back pain [20], chronic cardiovascular disease, and psychological disorders [21]. Notably, while musculoskeletal disorders, including neck pain, have shown anassociation with DT [22], there exists a dearth of comprehensive investigations into the effects of this therapy on individuals afflicted with CNP. Furthermore, the efficacy of DT remains contentious, predominantly stemming from challenges related to treatment precision, variable methodologies, inadequately sized cohorts [23], suboptimal methodological rigor, and inconclusive dosing protocols in extant trials. Nonetheless, the feasibility of imparting DT, along with conventional neck exercises, to larger group settings merits consideration. Not only do group settings foster social support dynamics [24], but they also offer cost-effective alternatives to individualized treatments [25]. 

In light of the aforementioned challenges and the growing interest in DT’s potential for musculoskeletal disorders, including CNP, our study seeks to address these gaps in knowledge. This randomized controlled study aims to assess the efficacy of a 12-week DT intervention on various outcomes, including pain, physical function, mental well-being, and quality of life, while also exploring potential underlying mechanisms of modulation. Our hypothesis postulates that this particular exercise regimen holds the potential to alleviate neck pain in adult individuals. Furthermore, this investigation seeks to elucidate the mechanistic pathways through which DT may exert its pain-reducing effects.

## Methods

### Study design

**Fig. 1:**
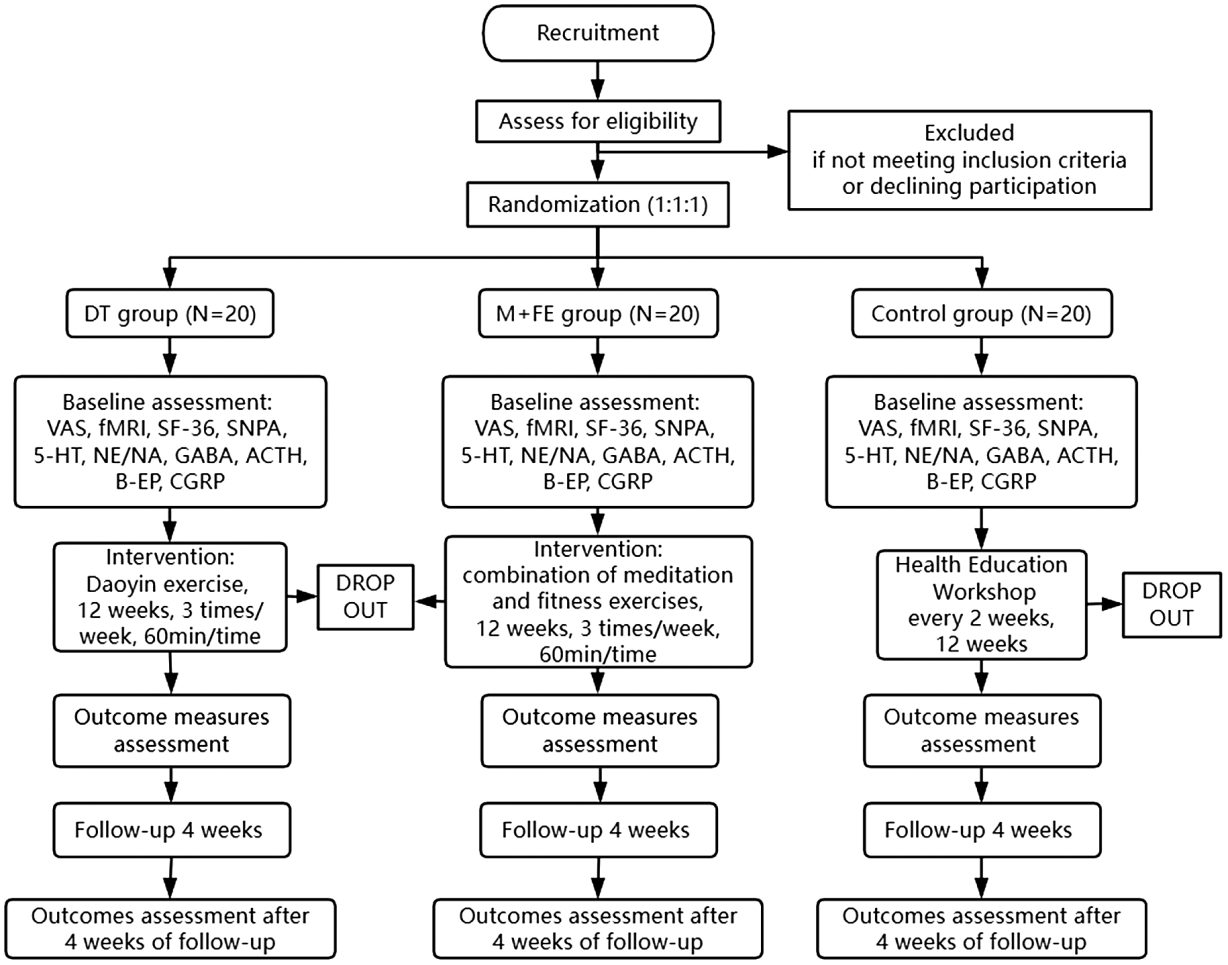
Trial flow chart. Diagram illustrating the randomized controlled trial protocol

This study represents an RCT with three distinct groups: the Daoyin Therapy group (DT intervention), the Meditation + Fitness Exercise group (M + FE intervention), and Control group. The Institutional Review Board approval was obtained from the Shanghai University of Sport (Project Number: 102772023RT016). We originally planned to recruit a total of 60 eligible individuals suffering from CNP who would then be randomly assigned to one of these three study arms, ensuring a balanced distribution among them. Prior to participating in the study, all participantswere required to provide written informed consent. The data collection and analysis for outcomes assessment will be carried out by independent researchers who are blinded to participant allocation. The primary treatment center for this trialis situated at Shanghai University of Sport. A schematic representation of the study design can be found in Fig. 1.

### Study population

The primary target for recruitment in this trial was individuals who were experiencing CNP persisting for more than three months, with no relief for a consecutive period exceeding two weeks. Interested individuals who expressed a desire to participate in the trial underwent a face-to-face interview, either conducted in a reception room within a community activity center or conducted online, depending on their preference. Individuals meeting the inclusion criteria were eligible to enroll in the trial after providing their informed consent through the signing of a consent form. To reach potential participants, we employed various recruitment strategies, including: (1) Placement of informative posters at affiliated hospitals of Shanghai University of Sport and nearby community centers; (2) Utilization of online platforms and digital channels to disseminate information and attract potential participants; (3) Collaboration with relevant agencies and partners to expand outreach efforts.

### Sample size

The sample size for this study was calculated using the G*Power software (version 3.1.9.7, University of Düsseldorf, Germany). The calculation was based on recent controlled trial studies related to exercise interventions for neck pain [26].Specifically, we employed the F test within the ANOVA framework with an effect size (Cohen’s f) set at 0.4, a significance level (α) of 0.05, and a desired statistical power (1 - β) of 0.8. The calculations determined that a minimum total sample size of 52 individuals was necessary for this study. To accommodate a potential 10% attrition rate, our recruitment target was set at 60 participants, evenly distributed across three groups, with a minimum of 20 participants per group. 

### Inclusion criteria

The inclusion criteria are: First, potential participants must fall within the age range of 18 to 65 years. They should also have been experiencing neck pain symptoms for a minimum duration of three months. Importantly, candidates should not have a history of prior shoulder or neck surgeries, and they should not present any concurrent shoulder issues. Furthermore, individuals interested in participating should possess a proficient understanding of the Mandarin language, enabling effective communication and the ability to read and write Chinese without any impediments such as dyslexia. In addition, prospective participants should not have been diagnosed with movement disorders and should demonstrate the capability to perform voluntary movements. Lastly, a vital criterion is the expression of a genuine willingness to actively engage and participate in the study, underscoring the importance of their commitment to the research endeavor. 

### Exclusion criteria

The exclusion criteria were individuals with a documented history of whiplash injury, head/neck injuries, or specific disorders affecting the cervical spine, such as disk prolapse, spinal stenosis, postoperative conditions, cervical radiculopathy, or myelopathy. This also extended to individuals with significant spinal deformities, neurological diseases, artificial joints, or metal implants. Additionally, individuals with severe cardiovascular disease or other medical conditions that contraindicated participation in physical activities were ineligible. Those with mental illness or an inability to independently complete the requisite questionnaires due to cognitive limitations were excluded. Participants who had undergone clinical treatment for neck pain in the past three months, were pregnant, had recently given birth, or could not speak or write in Chinese to complete the necessary questionnaires were not considered. Furthermore, individuals who had shown discomfort or aversion towards engaging in regular practice of Baduanjin, Yijinjing, Qigong, or Taichi were excluded. Lastly, individuals demonstrating poor cooperation or a lack of willingness to actively participate in the trial were not eligible for inclusion. 

### Dropout criteria

Participants who do not successfully complete the clinical protocol for specific reasons are classified as dropouts. These reasons include voluntary withdrawal by the participant, typically due to perceived treatment inefficacy or the emergence of adverse reactions. Participants are considered dropouts if they are lost to follow-up, rendering them inaccessible for necessary assessments. In some instances, researchers may opt to remove a participant from the study due to issues related to poor compliance with study protocols or in cases of serious adverse events that mandate discontinuation of their participation.

### Suspension criteria

Suspension may occur if significant safety concerns arise, requiring immediate investigation and mitigation measures. If the therapeutic effect is deemed inadequate, a simple physical assessment conducted at the fourth weekwould revealinsufficient efficacy of the trial. In cases of substantial deviations or critical errors in the trial plan, suspension may be necessary to rectify issues and ensure the study proceeds in accordance with the established protocols. 

### Randomization

The randomization process was conducted using Microsoft Excel. Prior to random assignment, the research team collected detailed participant information, including their name, date of birth, participant and center code, and date of inclusion. The randomization list was generated in Excel spreadsheet, where each participant was assigned a random whole number between 1 and 3. After sorting the random numbers, a column was numbered sequentially as “1, 2, 3, 1, 2, 3...” to correspond to the three groups: Group 1 (DT group), Group 2 (M + FE group), and Group 3 (control group). The random numbers were placed in an opaque envelope, which was sequentially numbered. This method ensured transparency and simplicity in the randomization process, with the random list securely stored in a password-protected computer accessible only to the designated researcher responsible for participant assignment. Staff and coaches did not have access to this information.

### Blinding

Participants were intentionally kept uninformed about the specific type of treatment they would receive throughout the course of the trial. Participants might not have effectively differentiated between DT and the other intervention, making the blinding feasible, except for participants in the control group, as they did not participate in any exercise interventions. A strict blinding protocol was maintained, encompassing outcome assessors, data managers, and the statistician. These key personnel remained unaware of the treatment assignments and did not share any study-related information among themselves. To uphold the blinding process, only the designated coaches possessed knowledge of the treatment allocations. It was imperative that the coaches, who were privy to this information, underwent training to effectively communicate with participants while maintaining the blinding of treatment assignments. This stringent blinding procedure was diligently upheld until the point at which the data were locked, ensuring that the integrity of the trial was preserved and that the results remained unbiased and reliable.

### Interventions

Participants in the DT and M + FE groups attended their respective training classes three times a week for 12 weeks. Coaches instructed and corrected movements during these sessions, teaching Daoyin, meditation, and fitness exercises. Instructional videos were provided to participants for home practice, and participants could send self-recorded videos to coaches via online communication platforms for feedback. Each DT or M + FE exercise classlasted 40 min continuously, excluding warm-up and relaxation time. Assessments were conducted at baseline, at the conclusion of the 12-week intervention period, and again at the 16-week.

Within each class of the DT group, participants underwent Daoyin exercise tailored for neck pain following a 3-step protocol. This protocol included a 10-min warm-up exercise session involving joint and basic Daoyin exercise to prevent sports injuries. The subsequent 40-min Daoyin exercise session comprised guided single, combination, and full-body movements. Finally, a 10-min relaxation exercise sessionfollowed to soothe and relax the body.

Within each class of the M + FE group, participants received a combination of meditation and fitness exercises designed to address neck pain, following a similar 3-step protocol. The warm-up sessionlasted for 10 min and involved joint exercises and basic limb movements. This was followed by a 40-min session consisting of 20 min of guided meditation activities and 20 min of single, combined, and comprehensive fitness exercises focused on neck pain. Lastly, participants engaged in a 10-min relaxation exercise session.

The control group did not receive any specific intervention but were instructed to continue their daily routines. However, participants in the control group were required to attend health education workshops every 2 weeks as part of their study participation.

### Primary outcome measurement

#### SC-NPAD

The simplified Chinese Neck Pain and Disability Scale (SC-NPAD) underwent a rigorous translation and validation process conducted by the Department of Orthopedics at Changhai Hospital, affiliated with the Second Military Medical University. The reliability and validity of the SC-NPAD were systematically assessed. The internal consistency, as indicated by Cronbach’s alpha (α), demonstrated high reliability levels. Furthermore, a factor analysis of the scale revealed its multidimensional structure, comprising four distinct factors: “pain” (items 1–3, 5, 7, 20), “disability” (items 4, 6, 8–11, 15), “neck function” (items 16–19), and “affective and cognitive influence” (items 12–14). The scale exhibited excellent repeatability, with Cronbach’s alpha values ranging from 0.770 to 0.977 for each item [5].

### Secondary outcome measurements

#### MRI

A 3.0T Magnetic Resonance Imaging (MRI) system, equipped with a 32-channel standard head coil, was employed in the imaging procedures at the key laboratory of Shanghai University of Sport. During the MRI scans conducted for the experimental task, participants were positioned in a supine posture and provided with specialized earphones designed to stabilize their heads and minimize the impact of instrument-related noise interference.

#### Blood tests

Blood samples of 8–10 ml were collected from each participant on multiple occasions. The levels of 5-Hydroxytryptamine (5-HT), Norepinephrine/Noradrenaline (NE/NA), and γ-aminobutyric acid (GABA) were quantified utilizing high-performance liquid chromatography (HPLC). The assessment of Adreno-Cortico-Tropic-Hormone (ACTH) was conducted through chemiluminescence immunoassay (CLIA), while β-Endorphin (β-EP) levels were determined using enzyme-linked immunosorbent assay (ELISA). Additionally, the evaluation of Calcitonin-Gene-Related Peptide (CGRP) was performed through radioimmunoassay (RIA). 

#### SNPA

The Static Neck Posture Assessment (SNPA) encompasses several key parameters, including the measurement of the forward head angle (FHA) and forward shoulder angle (FSA). Additionally, the assessment covers the range of motion (ROM) of the neck, which includes flexion, extension, left lateral flexion, right lateral flexion, left rotation, and right rotation.

#### VAS

The Visual Analogue Scale (VAS) assessment procedure entails drawing a 10 cm horizontal line on a sheet of paper. One end of this line is marked as 0, signifying the absence of pain, while the opposite end is marked as 10, representing severe pain. During the assessment, the participant was positioned facing the non-scaled side of the line, ensuring they aligned with the straight line. The participant was then instructed to mark a specific point on the line to indicate both the intensity of pain and the extent of their psychological sensation related to the pain. The healthcare provider, in turn, faced the scaled side of the line and measured the distance from the starting point to the participant’s marked position, which quantified the level of pain experienced. Clinical evaluation categorizes the VAS scores into three ranges: scores from 1 to 3 indicate “mild pain”, scores from 4 to 6 denote “moderate pain”, and scores ranging from 7 to 10 represent “severe pain” [27].

#### SF-36

In 1991, the Chinese version of the Short Form 36 questionnaire (SF-36) was translated by the Social Medicine Teaching and Research Office of the University School of Medicine. Subsequently, Chinese scholars conducted a comprehensive reliability and validity assessment of the Chinese SF-36 version, employing Cronbach’s alpha coefficient as a key metric to evaluate the scale’s internal consistency reliability. The quality of life scale analyzes eight dimensions: Physical Functioning (PF), Role-Physical (RP), Bodily Pain (BP), General Health (GH), Vitality (VT), Social Functioning (SF), Role-Emotional (RE) and Mental Health (MH). In addition to the SF and VT dimensions, the consistency reliability coefficients were computed for the remaining six dimensions, resulting in values ranging from 0.72 to 0.88, signifying good test-retest reliability (ranging from *r* = 0.70 to 0.94). Notably, the validity success rates for all dimensions, except SF, ranged from 75% to 100%, underscoring robust construct validity. Additionally, the discriminant validity success rates also demonstrated strong performance, ranging from 87.5% to 100%. These findings affirm the reliability and validity of the Chinese SF-36 version and support its utility as a comprehensive assessment tool for various dimensions of health-related quality of life [28].

### Safety evaluation

Participant safety was vigilantly monitored throughout each intervention. A team comprising one coach and two responsible assistants collaborated closely with clinicians during every class to provide guidance and ensure safety. In the context of this trial, adverse events are defined as occurrences that meet any of the following criteria: (1) result in severe pain; (2) induce dizziness, severe dizziness, or even syncope; (3) necessitate hospitalization; (4) impede the individual’s ability to work; (5) pose a threat to life. In the event of any adverse events, regardless of their association with the interventions, immediate cessation of treatment would be implemented. Participant experiencing adverse events would promptly receive appropriate remedial treatment. Furthermore, these adverse events would be reported to the relevant responsible entities and ethical committees for evaluation, with a decision made on whether the participant should discontinue participation in the trial. Participant encountering an adverse event would be subject to follow-up until the event has been resolved or until the participant’s condition achieves a state of chronic stability.

### Follow-up

To assess the short-term efficacy and safety of the intervention, participants underwent a 4-week follow-up period immediately after completing treatments. During this period, participantsdid not receive any additional treatment, and there was no direct researcher supervision. At the end of the 16-week period, participants were required to complete a third test. Furthermore, participants had the opportunity to communicate their clinical symptoms and report any adverse events through in-person discussions, text messages, or via online communication platforms at the designated time points.

### Data collecting and monitoring

Demographic and baseline characteristic data were gathered by outcome assessors at the time of participant recruitment. In order to maintain data accuracy, two independent data administrators performed data entry and conducted thorough proofreading. Once the data integrity was verified, the database was reviewed and confirmed by a qualified expert and two principal research members. Subsequently, the database was locked, with access restricted to the data administrators and the statistician.

### Statistical analyses

Demographic and baseline information will be compared among the different groups. The Kolmogorov-Smirnov test will be employed to assess the normality of data. For intragroup hypothesis testing, differences across various time points will be evaluated using repeated measures analysis of variance (RM-ANOVA) for normally distributed data, and Friedman’s test will be employed for data not following a normal distribution. In the case of intergroup hypothesis testing, normally distributed data will be analyzed using multivariate analysis of variance (ANOVA), while non-normally distributed data will be analyzed using the Kruskal-Wallis H test. Generalized Estimating Equation (GEE) model will be used to assess how the primary outcome measures, SC-NPAD, change over time across the three intervention groups. Covariates such as demographic factors, and other relevant variables will be included in the model to account for potential confounding effects. The GEE model will also be employed to examine the effects of the intervention on secondary outcome measures, such as SNPA scores for neck and shoulder posture and function, SF-36 scores for quality of life, and levels of neurotransmitters (5-HT, NE/NA, GABA, ACTH, β-EP, and CGRP) obtained from blood tests. Similar to the primary outcome analysis, this analysis will consider the longitudinal nature of the data and adjust for potential confounders. The GEE model will estimate the average change in outcome measures over time within each intervention group and to compare the differences in changes between the intervention groups and the control group. Interaction terms may be included in the model to examine whether the effects of the intervention vary over time or across different groups of participants. Appropriate adjustments for multiple comparisons will be made if necessary. Statistical significance will be taken at 2-sided *P* < 0.05. Statistical analyses will be performed using SAS 9.4 (SAS Institute Inc., Cary, NC).

### Ethical considerations

Participants retain the right to terminate their involvement in the study at any point, without any obligation. The findings of this study will be presented while ensuring the complete anonymity of individual participants, with no personal information disclosed. All data collected for this research will be solely utilized for the purposes of this study, and strict ethical principles of confidentiality are adhered to by all members of the research team. Proactive measures will be taken to address any ethical concerns that may arise during the course of the study. Ethical approval [No. 102772020RT112] has been obtained from the Institutional Review Board of Shanghai University of Sport. This trial has been registered with the Chinese Clinical Trial Registry (Registration ID: [ChiCTR2400079571]). Participants assigned to the control group were informed that they would receive one of three treatments (DT/M/FE) upon completion of the study. Further instructions regarding the timing and conditions for receiving these treatments will be provided to the participants.

## Discussion

Over the past decade, both European countries and the United States have issued comprehensive guidelines concerning the physical and non-pharmacological management of CNP. Notable examples include the 2008 guidelines from the International Panel of Experts on chronic pain management, the 2010 practice guidelines from the American Society of Anesthesiologists (ASA) on chronic pain management, and the 2013 guidelines from the US Institute for Clinical Systems Improvement (ICSI) regarding the assessment and management of chronic pain. Furthermore, in 2018, the Royal Dutch Society for Physiotherapy (KNGF) released clinical practice guidelines tailored to physical therapists, aiming to provide recommendations for the diagnosis and treatment of CNP. Similarly, the American Physical Therapy Association (APTA) published the 2017 edition of Clinical Practice Guidelines for CNP, while Canada issued the Clinical Practice Guidelines for Neck Pain and Related Disorders in 2016 and the Clinical Practice Guidelines for the Nonpharmacological Treatment of Headache and Neck Pain in 2019. These guidelines collectively address a wide spectrum of health, social, and economic aspects associated with neck pain, emphasizing the importance of non-pharmacological treatments. In 2019, a Chinese expert group also collaborated to release the Chinese Expert Consensus on Exercise Therapy for Neck Pain, which is tailored to Chinese characteristics and seeks to establish norms and recommendations for exercise therapy in the context of neck pain. These guideline outcomes substantiate the feasibility of implementing their recommendations, highlighting the availability of non-pharmacological treatments for chronic pain that exhibit sustained effectiveness beyond the treatment period. Notable interventions include exercise, cognitive-behavioral therapy, multidisciplinary rehabilitation, and mind-body interventions. Additionally, some complementary and integrative medicine therapies, such as acupuncture and chiropractic care, have demonstrated sustained effects on function post-intervention, albeit with limited evidence beyond the 12-month mark. Importantly, there is no substantial evidence of serious harm associated with these interventions, although data on potential harm are limited.

According to the evidence levels outlined by the Oxford Centre for Evidence-Based Medicine, a diverse range of exercise therapy techniques is strongly recommended for the management of CNP. These include plyometric training, which comprises deep neck muscle training and shoulder-neck plyometric training, aerobic exercise/endurance training, core stability training/motor control involving cervical spine stability training and shoulder stability training, stretching training/flexibility training, muscle energy techniques, stress biofeedback training, breathing training, virtual reality training, as well as practices like Taijiquan, Yoga, Qigong, Baduanjin, Pilates, and integrated movement training. A meta-analysis reviewed 10 randomized controlled trials and found compelling evidence that exercise interventions significantly improved pain levels and dysfunction indices in individuals with CNP when compared to other treatment modalities [29]. Additionally, another study provided evidence supporting the effectiveness of deep neck muscle training in ameliorating symptoms among patients with CNP [30]. Furthermore, an investigation furnished evidence that Qigong can yield improvements in pain levels and dysfunction indices in individuals suffering from neck pain [31]. These findings collectively underscore the efficacy and utility of various exercise-based interventions in addressing CNP, offering a range of options for patients and healthcare providers to consider in treatment planning.

Daoyin holds a significant place within TCM and has made substantial contributions to the health and well-being of individuals in China over thousands of years. According to TCM principles, a healthy human body relies on the harmonious coordination of internal organs and the balance of qi and blood. Daoyin, as a practice, embodies the principles of body, breath, and mind harmony. It places particular emphasis on the fusion of physical strength and meditative aspects, employing a combination of static postures and dynamic movements. This practice is designed to facilitate the circulation of vital energy and blood, strengthen muscles, nourish tissues and organs, and alleviate muscle spasms. Prior research has indicated that consistent and long-term engagement in Daoyin training can lead to increased skeletal muscle strength, enhanced motor function, improved activities of daily living, and positive effects on mental well-being [32]. While a substantial body of scientific evidence supports the efficacy of such therapies for promoting human health, their specific effectiveness in the context of neck pain remains to be established. Therefore, it is of paramount importance to generate robust evidence regarding the efficacy of Daoyin as a potential intervention for CNP. Our proposed RCT seeks to contribute to the understanding of Daoyin’s role in addressing CNP by providing empirical evidence to support its application in clinical practice.

Presently, studies have employed various methodologies and imaging techniques to elucidate the impact of CNP on both the peripheral and central nervous systems. These investigations aim to uncover the associations between structural and functional changes in the brain and alterations in pain perception, all within the context of clinical application. However, there exists a notable gap in research that systematically investigates the influence of brain plasticity, influenced by diverse treatments, on individuals with CNP. Our proposed RCT extends to exploring the intrinsic connections with various clinical symptoms, changes in the disease progression, and potential mechanisms underlying these phenomena. Specifically, there is a scarcity of studies that delve into the realm of non-pharmacological treatments, particularly exercise therapy, and their impact on brain plasticity in individuals with CNP. The current body of literature has yet to comprehensively address these aspects, leaving a significant knowledge gap in our understanding of how different treatments may affect brain plasticity, clinical symptoms, and the overall trajectory of the disease. As such, further research in this domain is warranted to shed light on these intricate relationships and mechanisms.

This article provides a comprehensive account of an RCT designed to assess the outcomes related to pain, specific characteristics, and structural functions of brain activity, the neuroendocrine system, quality of life, and postural stability. The trial aims to evaluate the efficacy of distinct treatments for CNP. Furthermore, the study offers the opportunity to delve deeper into the mechanisms underpinning pain improvement through the practice of DT.

Pain represents the paramount symptom in the context of CNP assessment. Therefore, the primary outcome measure in our proposed RCT is SC-NPAD, which effectively gauges the intensity of neck pain. Among the secondary outcomes, SNPA measures the active range of motion of the neck using a straightforward approach. MRI is utilized to assess the impact of DT on enhancing brain structure and function in individuals experiencing pain. VAS is also employed to assist in evaluating pain intensity. Furthermore, the study employs SF-36 to assess the participants’ quality of life. To gain insights into the regulatory mechanisms of the neuroendocrine system contributing to pain alleviation, the study observes the levels of transmitter content in pain-related neural pathways. This includes 5-HT, CGRP, GABA, β-EP, ACTH, and NE/NA. By utilizing these outcome measures, the study aims to comprehensively evaluate the efficacy of DT in managing CNP while also shedding light on the underlying mechanisms involved.

The designed RCT and its rigorous methodology will facilitate the collection of valuable and high-quality data, which will be instrumental in evaluating the effectiveness of Daoyin and meditation + fitness exercise in the management of CNP. This trial is poised to make a substantial contribution to establishing a robust foundation for the clinical treatment of CNP, while also offering insights that can inform future research endeavors pertaining to DT and M + FE as therapeutic modalities for CNP or other diseases.

## Trial status

Participants were recruited from March to May 2023. Study completion is expected to be March 2024.

## Data Availability

Yes

## Abbreviations

CNP: Chronic neck pain
DT: Daoyin therapy
M + FE: Meditation + fitness exercise
TCM: Traditional Chinese medicine
SC-NPAD: Simplified Chinese-neck pain and disability
VAS: Visual analogue scale
MRI: Magnetic resonance imaging
SF-36: 36-item short-form health survey
SNPA: Static neck posture assessment
FC: Functional connectivity
fMRI: Functional magnetic resonance imaging
NMDA: N-methyl-D-aspartic acid receptor
RVM: Rostral ventromedial medulla
SERT: Serotonin transporter
PAG: Periaqueductal gray
5-HT: 5-Hydroxytryptamine
NE/NA: Norepinephrine/noradrenaline
GABA: γ-aminobutyric acid
HPLC: High-performance liquid chromatography
ACTH: Adreno-cortico-tropic-hormone
CLIA: Chemiluminescence immunoassay
β-EP: β-Endorphin
ELISA: Enzyme-linked immunosorbent assay
CGRP: Calcitonin-gene-related peptide
RIA: Radioimmunoassay
FHA: Forward head angle
FSA: Forward shoulder angle
ROM: Range of motion
APTA: American Physical Therapy Association
GEE: Generalized estimating equation
PF: Physical functioning
RP: Role-physical
BP: Bodily pain
GH: General health
VT: Vitality
SF: Social functioning
RE: Role-emotional
MH: Mental health
